# Elephants know when their bodies are obstacles to success in a novel transfer task

**DOI:** 10.1038/srep46309

**Published:** 2017-04-12

**Authors:** Rachel Dale, Joshua M. Plotnik

**Affiliations:** 1 Think Elephants International, Stone Ridge, NY, USA; 2 Dept. of Psychology, University of Cambridge, Cambridge, UK; 3 Mahidol University–Kanchanaburi, Sai Yok, Kanchanaburi, Thailand; 4 Golden Triangle Asian Elephant Foundation, Chiang Saen, Thailand

## Abstract

The capacity to recognise oneself as separate from other individuals and objects is difficult to investigate in non-human animals. The hallmark empirical assessment, the mirror self-recognition test, focuses on an animal’s ability to recognise itself in a mirror and success has thus far been demonstrated in only a small number of species with a keen interest in their own visual reflection. Adapting a recent study done with children, we designed a new body-awareness paradigm for testing an animal’s understanding of its place in its environment. In this task, Asian elephants (*Elephas maximus*) were required to step onto a mat and pick up a stick attached to it by rope, and then pass the stick forward to an experimenter. In order to do the latter, the elephants had to see their body as an obstacle to success and first remove their weight from the mat before attempting to transfer the stick. The elephants got off the mat in the test significantly more often than in controls, where getting off the mat was unnecessary. This task helps level the playing field for non-visual species tested on cognition tasks and may help better define the continuum on which body- and self-awareness lie.

Self-awareness is a seemingly rare capacity in the animal kingdom due to the complexity of the cognition that most likely underlies it^1^, and the limitations of testing for it in controlled settings^2^. Specifically, self-awareness suggests an understanding or recognition of the self that is often linked to more complex forms of perspective taking and empathy^1^,^3^,^4^,^5^. In addition, the capacity for self-awareness suggests an individual can separate the ‘self-entity’ from the ‘other-entity’, and represents the “ability to become the object of your own attention” (ref. 6, pp.9). The most recognised and often-used test of self-awareness is the mirror mark or mirror self-recognition (MSR) test^7^. In this experiment, an animal’s behaviour in front of a mirror is interpreted as self-recognition only if the animal progresses through specific behavioural stages over a particular time period of mirror-exposure (see ref. 8 for a procedural review). These stages usually begin with an animal displaying social behaviour (i.e., treating the mirror reflection as if it were a conspecific) when it is first exposed to its mirror reflection. In fact, most animal species tested on this capacity for mirror self-recognition never move beyond this stage^8^, continually treating the mirror image as if it were a stranger (or, in some cases, a friend-ref. 9). Those species that move beyond social behaviour usually do so within a few hours or days of mirror exposure, when they begin to display behaviour directed toward their own bodies^7^,^10^. This behaviour often includes inspection of otherwise hard-to-see places, close-eye inspection, or facial grooming^8^. When observed during a controlled experiment, this behaviour is often taken as an indication of ‘mirror self-recognition’ or the animal’s understanding that the mirror image is actually a reflection of its own body^7^. As the experimenter’s job of defining these behaviours as “self-directed” is inherently subjective, an objective ‘mark’ test is performed whereby the animal is marked inconspicuously on their head prior to mirror exposure. If the animal touches the mark on their own body in this condition, but not during conditions that control for other, non-visual mark-related cues, it passes the test. Such performances have been claimed to demonstrate that the animal recognises itself in the mirror, and so is self-aware^1^,^7^.

Outside of humans, MSR has thus far only been demonstrated in a few species, including the other great apes^6^,^7^,^8^, but not monkeys^11^,^12^. Povinelli & Cant^13^ proposed this may be because great apes require an awareness of their large bodies as they move through trees, whereas the small bodies of monkeys allow them to use stereotyped movements. However, this “arboreal clambering hypothesis” may no longer hold due to subsequent demonstrations of MSR in dolphins^14^, elephants^10^, and magpies^15^.

The mirror self-recognition test has its critics, however, with some arguing it is not an adequate test of self-awareness nor is it linked to the capacity for empathy, and that the test itself is limited in its ability to investigate complex cognition^16^,^17^. These arguments stem from Gallup’s^1^ original assertion that the mirror test is a “marker of mind,” by which an animal that is able to understand the cognition of others must first have a concept of self. Although Gallup^1^,^18^ has argued that this is an all-or-nothing capacity, others have argued that self-awareness is represented by a continuum within the animal kingdom, whereby some species have it, some do not, and some lie somewhere in between (eg., refs 9,14, 19 and 20).

Regardless of how one attempts to interpret the cognition underlying self-recognition, the demonstration of MSR in a select-few species is most interesting because of its seemingly consistent link to other, more complex social traits^1^. Indeed the species that have demonstrated MSR all show varying levels of cooperative problem-solving, perspective taking and empathy (e.g., chimpanzees: refs 21, 22, 23, corvids: for a review, see ref 24, 25; elephants: refs 26 and 27; dolphins: for a review, see ref. 28), suggesting that self-awareness may relate to effective cooperative-living in socially intelligent animals. The link may become more apparent with further research on the understanding animals have about the distinction between one’s self, others, and their environment. A more developed self-understanding of how an individual relates to those around it may underlie more complex forms of empathic perspective taking and targeted helping directed at others in need, as seen in studies on human children^29^,^30^.

This link between the self-other distinction (as measured by MSR) and other-regarding behaviour (like targeted helping or empathetic behaviour^23^) becomes clearer as psychologists develop new tests for identifying each. Although the latter is investigated using a variety of paradigms (e.g., observational studies: children^31^; chimpanzees^32^,^33^; elephants^34^, helping tasks: children^35^; chimpanzees^36^,^37^, consolation research: children^29^; chimpanzees^38^; elephants^27^), the former has, to date, only been studied using the MSR test. Failure to show mirror self-recognition in some species may be indicative of species-related limitations in gathering perceptual information from a purely visual mirror apparatus^39^,^40^,^41^, issues with the salience of the mark itself, or differences in individual or species-level interest in one’s own body rather than a lack of self-other understanding.

One potential complement to the mirror test as a measure of self-understanding may be a test of ‘body-awareness’. This test specifically looks at how individuals may recognise their bodies as obstacles to success in a problem-solving task. Importantly, success on such a ‘body-awareness’ task would not require visual recognition of an image, or a desire to investigate novel markings on the body. Such a task could demonstrate an individual’s understanding of its body in relation to its physical environment, which may be easier to define than the self-other distinction demonstrated through success on the mirror test. Thus, body-awareness tests could nicely complement the mirror test in species that have already passed it, or be a good starting point for those species not yet tested in front of a mirror or those disadvantaged by the highly visual MSR test.

In one such body-awareness task designed by Moore and colleagues^42^ and Brownell *et al*.^43^ for human infants, participants were given a shopping cart to push. A mat was then attached to the back of the cart and the child was instructed to push the cart to their caregiver. Because the child could not reach the handle without first standing on the mat, passing the task required that the child realize that his or her body was an obstacle to success and that he or she needed to step off and to the side of the mat to push the cart. Moore *et al*.^42^ reported that, after controlling for age, efficiency in solving the body self-awareness task was significantly positively correlated with success on the mirror mark test. This suggests that body-awareness and self-awareness may share space on the same continuum of self-understanding, although clearly further research is needed. In this study, we adapted the shopping cart task for use with a non-human animal, the Asian elephant (*Elephas maximus*), to investigate whether a non-human species that had previously passed the MSR test would also pass a body-awareness task. To adapt the test with infants for elephants, we removed the need to push an artificial object and replaced it with the need to pull and transfer a stick (Supplementary Movie S1). We attached a stick to a rubber mat using a rope; the elephants were then required to walk onto the mat, pick up the stick and pass it to an experimenter standing in front of them (Fig. 1). Specifically, we investigated whether elephants understood the role of their bodies as potential obstacles to success in the task by observing how and when elephants removed themselves from the mat in order to exchange the stick.

In our experiment, we also instituted control conditions designed to differentiate between behaviour indicative of a self-obstacle relationship and other, more cognitively parsimonious reasons as to why the elephants might step off the mat while performing the task. We conducted two control conditions. The first was identical to the experimental condition except that the stick was not attached to the mat and thus subjects did not need to get off the mat in order to pass the stick. This controlled for any prior conditioning or learning that may have influenced the elephants’ tendency to get off the mat regardless of whether or not it was required for successful completion of the task. We included a second control condition to rule out the possibility that the elephants’ tendency to get off the mat was due to discomfort felt under foot during the course of a trial and, more specifically, when the subject pulled on the stick in the test. In this condition, an experimenter pulled on the rope attached to the mat to create a tug of force underfoot while the elephants again performed a transfer, but this time from right to left rather than forwards. Thus, it was again not necessary for the elephants to get off the mat to complete a trial successfully. We predicted that elephants would demonstrate body-awareness in this task by removing themselves from the mat during test conditions in which the stick was attached to it, but not in control conditions where removing one’s body from the mat was unnecessary.

In general, elephants are regarded as large-brained, intelligent social mammals even though our empirical understanding of the cognition underlying their physical and social lives remains limited^44^,^45^. Given previous instances of success on the self-recognition test in Asian elephants^10^, and high levels of success during pilot work of the body-awareness task with captive African elephants (*Loxodonta africana*) in South Africa^46^, we hypothesized that Asian elephants would succeed on a task of body-awareness. Both MSR and body-awareness experiments aim to assess an animal’s understanding of its place within its natural environment, and may indicate different points along a self-understanding continuum. Success on the body-awareness task would, at least, suggest that more species-appropriate cognitive tests might lead to a more comprehensive approach to studying self-understanding in animals.

## Results and Discussion

### When did elephants get off the mat?

Because getting off the mat was only necessary for success in the test condition, we predicted that subjects would get off the mat more in the test condition than in either control condition. To assess whether or not the elephants got off the mat (1/0) in each condition, we ran a binomial GLMM. Condition and age group (young: 4–20 years, N = 7, old: 20–44 years, N = 5) were included as fixed effects, and subject identity and the order in which the conditions were presented were included as random effects (Tables 1 and 2). Wilcoxon signed-rank tests were run to investigate the main effect of condition post-hoc. There was no main effect of age on the likelihood (1 vs. 0 per trial) that subjects would get off the mat (N = 12, F_1_ = 0.17, p = 0.66). There was, however, a significant effect of condition (Table 1). Wilcoxon signed-rank tests, with a Bonferroni correction applied (*p* < 0.0125), revealed that subjects were significantly more likely to get off the mat in the test (mean = 42.33/48, SE ± 3.01) than in the unattached stick condition (mean = 3.25/48, SE ± 0.91, N = 12, *V* = 78, *p* = 0.002) and in the test than in the foot discomfort condition (mean = 12.08/48, SE ± 4.93, N = 12, *V* = −73, *p* = 0.008, Fig. 2 and Supplementary Data).

There was no significant difference between the two control conditions (N = 12, *V* = 18.5, *p* = 0.116). This demonstrates that overall, the subjects were significantly more likely to get off the mat in the test condition than in either of the two control conditions (Fig. 2).

### How did elephants perform in the first session of the test condition?

We conducted a relatively large number of trials per individual due to our small overall sample size. However, this may have resulted in learning over time. Therefore, we also separately considered the first test session. All elephants succeeded at least once in the first session of the test condition (12 trials, Fig. 3), with 8/12 elephants successfully getting off the mat and exchanging the stick in at least 11 of 12 test trials and six subjects showing success in their first trial. Furthermore, as a group, subjects performed above chance in their first session (mean = 9.5 successful trials out of 12, binomial test for 50% probability, p < 0.001), suggesting learning over time was not an important factor in determining the elephants’ overall success in this task.

### How did elephants perform in the control conditions?

Although the elephants got off the mat in significantly more trials in the test than in the control conditions overall, we also looked at whether the difference was due to learning. Thus, the first session (12 trials) of the test and the stick-unattached control and the test and the foot discomfort conditions were compared to each other using a binomial GLMM with condition included as a factor and subject and testing order as random effects (Table 1). When comparing the test and the stick-unattached control, results show that even in the first sessions, subjects got off the mat significantly more in the test (mean = 9.5/12, SE ± 0.98) than in the stick-unattached control (mean = 2.25/12, SE ± 0.72, χ^2^ = 82.08, P < 0.0001). Likewise, when considering the first session of the foot discomfort as compared to the first test session, subjects were more likely to get off the mat in the first test session than the first foot discomfort session (mean = 4.4/12, SE ± 1.41, χ^2^ = 47.25, p < 0.0001, Fig. 4). These results suggest that from the first session, the elephants recognised that the control condition tasks could be completed without getting off the mat.

To our knowledge, this study provides unique evidence of ‘body-awareness,’ as demonstrated using an experimental paradigm, in a non-human animal. The results show that Asian elephants are able to recognise their own weight as an obstacle and thus remove themselves from a mat in order to complete a task. In addition, they do so significantly more often in test than control trials, the latter of which do not require that the elephants get off the mat in order to succeed.

We recognise the potential for an order effect across the one test and two control conditions, and thus controlled for order by placing the elephants into three different groups in which the presentation order of the three conditions was different. Although we were unable to include order as a factor in the GLMM due to the low sample size in each grouping (N = 4), when controlling for it by including it as a random effect in the model (N = 12), we found that the differences between how often elephants got off the mat in test and control conditions remained statistically significant.

The tendency of elephants to get off the mat during the test condition might be explained by alternatives to the body-awareness hypothesis. Firstly, it is possible that the elephants’ tendency to get off the mat in the test condition was due to discomfort felt underfoot during the elephants’ tugging of the stick and rope. This might have lead subjects to get off the mat and thus discover the solution as a by-product of the initial discomfort. However, elephants got off the mat significantly less in the aforementioned foot discomfort control than in test trials, the former of which involved an experimenter tugging on the rope as hard as possible and at a similar angle to the elephants’ pull whilst the elephants performed a task where it was possible to stay standing on the mat. We recognise that a human cannot pull with the same force as an elephant. However, because of extensive discussions with the elephants’ permanent handlers and veterinary staff about the sensitivity of the elephants’ footpads^47^, we believe that if the mat were the cause of discomfort underfoot when the attached stick was tugged, this discomfort would be created with any force of pulling. We were originally more concerned that the elephants might have mistaken the experimenter’s pulls as a signal to get off the mat because of their prior, extensive experiences responding to human cues, but the results show that this was not the case.

In the stick-unattached control, subjects also removed themselves from the mat significantly less often than in test trials, demonstrating that they did not get off the mat in a control task nearly identical to the test a) when it was not necessary to do so, and b) simply because they had learned a contingency between their body’s place on the mat and success in the task. In other words, the elephants understood that although their bodies’ place on the mat was an obstacle to success in test trials, it was not in the stick-unattached control. Perhaps the most crucial group of elephants with regards to this condition was that which received the test before the stick-unattached control. There were eight subjects which received this testing order and the mean number of trials these subjects got off the mat in the stick-unattached control was 3.87 out of 48. This low mean is similar to that calculated from the four subjects which received this control before the test (2/48 trials), suggesting that experience on the test did not create a learned pattern of behaviour which resulted in the subjects always moving off the mat. This further supports our interpretation that the subjects recognised when it was, and was not, necessary to get off the mat to succeed on the task.

We cannot rule out the possibility that they may have removed their weight or repositioned their bodies off the mat in order to gain better positioning for moving the stick. Unlike in the studies with human children^43^,^48^, we were unable to perform a control condition in which a heavy weight was put on the mat instead of the subject’s body, nor were we able to perform a control in which the stick was weighted by a heavy object independent of the mat. These controls would have allowed a comparison of the elephant’s behaviour when either their body or another object was the obstacle preventing stick transfer. However, due to the size and strength of elephants, it was not possible to find an object heavy enough to serve as a suitable comparison in either control.

Although our sample size prohibited a comprehensive assessment of developmental differences in the emergence of body-awareness, there was no effect of age on the likelihood to get off the mat in any condition when our data were subjected to the model. This suggests that by four years of age the capacity for body-awareness may have already developed in elephants. It would be interesting for future studies to test especially young elephants to assess at what age this ability emerges. Human children develop body-awareness at the same age as mirror self-recognition, around 18–24 months^42^,^43^,^48^. Elephants have a similar life span to humans and it may be that parallels also exist in the ontogenetic timeline of self-awareness development. Due to this life cycle similarity, elephants would serve as an excellent model for a comparative approach to the development of self- and body-awareness.

In order to investigate whether there is a relationship between self-awareness as demonstrated by tests of mirror self-recognition (e.g. ref. 6) and success on a body-awareness task such as the one performed in this study with elephants, and with children^42^,^43^,^48^, further studies on children and other MSR-capable species (including elephants, magpies, dolphins and the great apes) are needed. In addition, due to the controversy over the cognitive implications of mirror self-recognition^16^,^17^, an investigation of body-awareness using a variation of the task employed in this study with traditionally MSR-incapable species might begin to lend credence to the theory that self-awareness exists as a continuum rather than as an all-or-nothing capacity^9^,^19^,^20^. This is a hypothesis already well-defined in the developmental literature to explain ontogenetic changes in an infant’s concept of self^49^,^50^,^51^,^52^. Elephants have shown success on both body- and self-awareness tasks tested to date, as demonstrated by Plotnik *et al*.^10^ and the current study, but it may also be that some species are successful on only one or neither. By examining the social and environmental niches of the animals that do and do not pass different tasks, we can gain greater insights into the convergent or phylogenetic origins of self-understanding.

By itself, MSR itself may not be a sufficient test of self-awareness in animals that lack acute vision, and thus body-awareness–which does not require a strong visual sense - may help parse out those species that should receive greater attention in cognition research. As Bekoff and Sherman^19^ have pointed out, progress in comparative self-awareness research has been limited until now by a focus on visual cues and this reliance may have led to false negatives on the MSR test. Many species, including elephants^45^ rely on chemical, olfactory or auditory cues for negotiating the world around them, including individual recognition of conspecifics. A body-awareness test may thus be a good first step in the further development of species-specific self-understanding studies that remove the human-centric advantage that limits the success of so many non-human animals on cognition tasks in general.

In conclusion, this study suggests that Asian elephants have the capacity for recognising that their bodies may serve as potential obstacles in the environment and so may have some specific awareness of the relationship between the self and external objects. Furthermore, self-understanding may express itself on a species-level continuum which includes, but is likely not limited to, self-awareness as demonstrated by MSR tests, and body-awareness as demonstrated by the test employed in the current study. Clearly, there is a need for greater variability in the ways in which psychologists assess non-human animals’ understanding of self. Specifically, greater attention is needed to the development of more novel, comparative and ecologically valid approaches that take the evolutionary strengths–size and sensory in particular – of individual species into account.

## Methods

### Subjects

We tested twelve elephants at the Golden Triangle Asian Elephant Foundation (GTAEF) in Chiang Rai, Thailand from November, 2012 to March, 2013 in 30-minute blocks from 0800–1030 and 1330–1530 hrs. The subjects live on the properties of the Anantara Golden Triangle Elephant Camp and Resort and the Four Seasons Tented Camp, and are cared for by their individual mahouts (elephant handlers). Elephant subjects have undergone extensive training for hotel guest programs (e.g., riding, trekking and mahout training courses), but had no previous training with a task similar to the one employed here. The mahouts and the full-time veterinary staff oversee the elephants’ daily husbandry regimes to ensure a high level of welfare. All subjects were female and ranged in age from 4 to 40 years old. Participation in all aspects of this test was voluntary and if at any point the subjects ceased to participate, the session was terminated. However, due to the elephants’ extensive training to interact with humans and because non-invasive experimental research is rewarding for the elephants, subjects were almost always enthusiastic to participate.

### Ethical Approval Statement

Our non-invasive work on elephant behaviour and cognition in Thailand was permitted by the National Research Council of Thailand [111/55]. The University of Cambridge Zoology Animal Use Committee reviewed and approved our non-invasive behavioural research protocols for Thailand [Z003/2011; Z003/2013], and this study’s methods were carried out in accordance with the guidelines and regulations of both aforementioned institutions.

### General Materials

A large rubber, light grey mat was cut to measurements of 200 × 115 cm, and a rope was attached halfway along the end of one short side using a carabineer. The rope measured approximately 100 cm long, but varied slightly across elephants due to the variability in their individual sizes and trunk reach. At the end of the rope, a stick was attached and placed parallel to the rubber mat on the ground. Trials were recorded on an SD card using a standard Sony digital video camera.

### Training

Subjects were first trained to stand on the rubber mat to familiarize them with a novel object. They were instructed to first walk in a straight line onto the mat. If they remained on the mat for at least 10 seconds, they were then permitted to freely leave the mat in any direction, without any instruction from the mahouts. If an elephant left the mat prior to the end of the trial, they were simply asked by the mahout to move back onto the mat. Training continued until the elephants completed 10 trials. In order to pass the experimental task, subjects were required to move off to the side of the mat before handing the stick to the experimenter or mahout, so it was important that initial training allowed the elephant to leave the mat in any direction voluntarily. All subjects stood on the mat without any apparent apprehension or difficulty and there was no apparent pattern in the direction they tended to move off the mat during this training period.

Subjects were also trained, in the absence of the mat, to walk up to a stick from three meters away, pick it up and give it to a familiar experimenter a further two meters away. All subjects already knew a command for picking up an object. Training initially involved commands and food rewards but once these were no longer needed, sets of 10 trials were run until subjects reached criterion (successfully passing the stick to the experimenter in five consecutive trials). For each subject, the same familiar experimenter (the first author, R.D.) was used throughout training and testing.

### Test and control conditions

The order of the three conditions (one test and two controls) was counterbalanced across subjects in order to control for the potential effect that experience may have on performance in subsequent conditions. Due to the limited number of subjects, a full counterbalance was not possible. Therefore, because the most important factor was the influence of the control conditions on performance in the test, subjects were divided into three groups of four elephants each, such that the test condition was either first or preceded by one of the control conditions. The effects of counterbalancing were controlled for in the GLMM analyses conducted on the data.

### Test Condition

**Figure 1:**
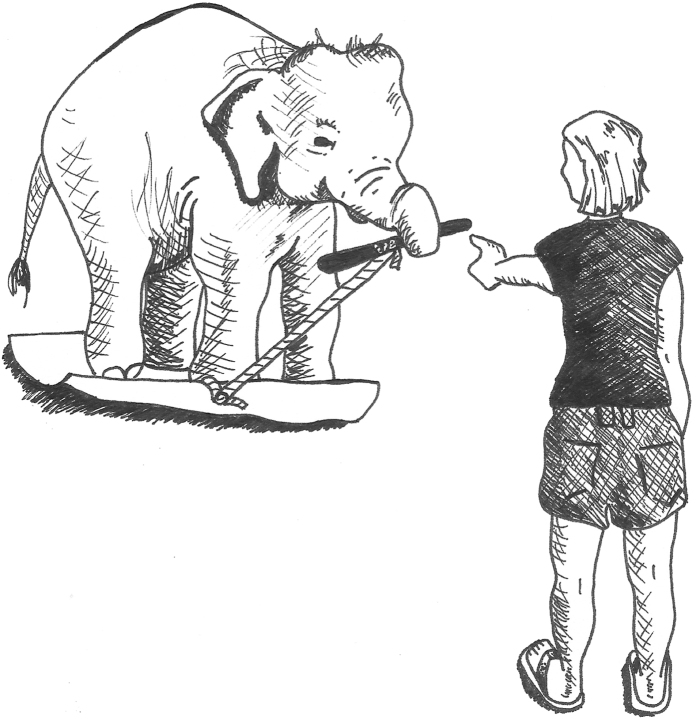
Set-up of test trials. Once standing on the mat, elephants were instructed to pick up the stick and give it to the experimenter. Drawing by E. Gilchrist.

**Figure 2:**
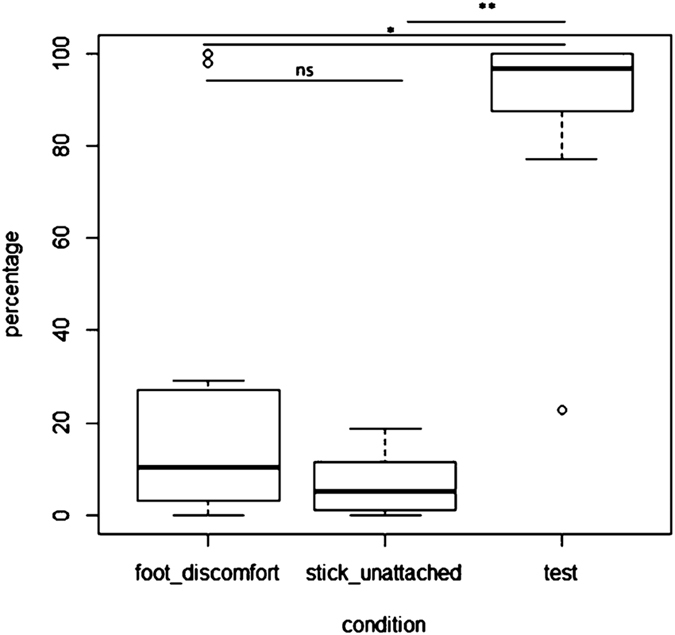
Percentage of trials subjects fully removed their weight from the mat in each condition. Boxplots represent 25th and 75th percentiles, centre line indicates the median, whiskers represent non-outlier range and dots are outliers. Significance levels: ** < 0.005, * < 0.01, ns = non-significant.

**Figure 3:**
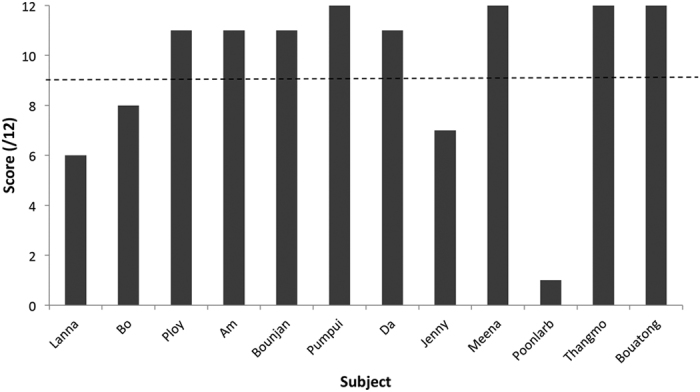
Individual performance of each subject on the first test session. The line represents statistical significance above chance level.

**Figure 4:**
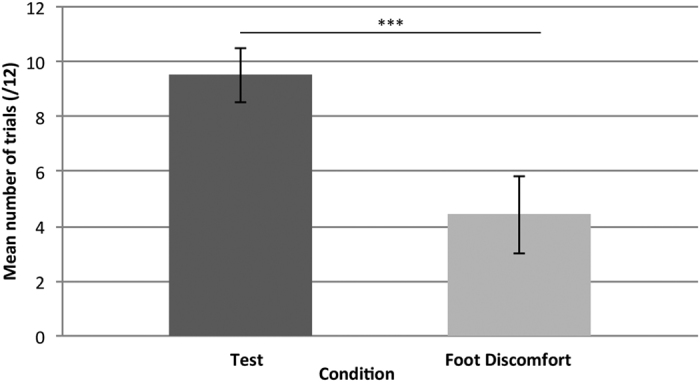
Mean number of trials [/12] subjects got off the mat in the first session of the test and foot discomfort conditions. *** < 0.0001.

Subjects began each trial at a distance of three meters away from the end of the mat. They were given the command to ‘go’ and allowed to walk onto the mat; subjects were required to have at least one foot on the mat when they picked up the stick or a trial was restarted. Once on the mat, the mahout, standing away from the subject and to the side of the experimenter, instructed the subject to pick up the stick and give it to the experimenter (Fig. 1). The trial ended when the subject stepped off the mat and pulled the stick and mat forward (success), after one minute of continuous attempts without success, or when the subject walked away and ceased to participate. Mahouts were present at all times during research with the elephants for safety reasons. Although it is possible that subtle cues by the mahouts may have affected the subject’s performance, this is unlikely given evidence that Asian elephants do not appear to follow clear visual cues provided by mahouts^40^ in social situations but instead seem to rely heavily on their mahouts’ vocal cues. We strictly controlled the vocal cues given by the mahouts during trials so that only ‘go’, ‘pick up’ and ‘come’ were used. See Movie S1 for video examples of all conditions.

### Control Condition 1: The Unattached Stick

In this condition, we investigated whether elephants knew when it was, and was not, necessary to get off the mat in order to successfully exchange the stick. We removed the connection between the rope and the mat so that the condition was identical to the test condition except that the elephant did not need to move off the mat in order to succeed in passing the stick to the experimenter. Subjects could continue moving forward and were able to leave their back two feet on the mat while passing the stick. The elephants’ front two feet needed to be off the mat due to the distance between the mat and the experimenter. Thus, we were able to keep the distance between the elephant and the experimenter consistent between the control and the test, while manipulating the fact that the elephant’s body was no longer an obstacle to success. If subjects recognised when their body was and was not an obstacle and had not simply learned an association between their body, the mat and the stick exchange, we would expect them to remove themselves from the mat in significantly more test than control trials.

### Control Condition 2: Foot Discomfort

We were also interested in controlling for the possibility that subjects did not like the initial feeling of the mat being tugged beneath their feet as they pulled on the rope in the test condition. If this was the case, they might have removed themselves from the mat due to discomfort rather than an understanding of the task.

**Figure 5:**
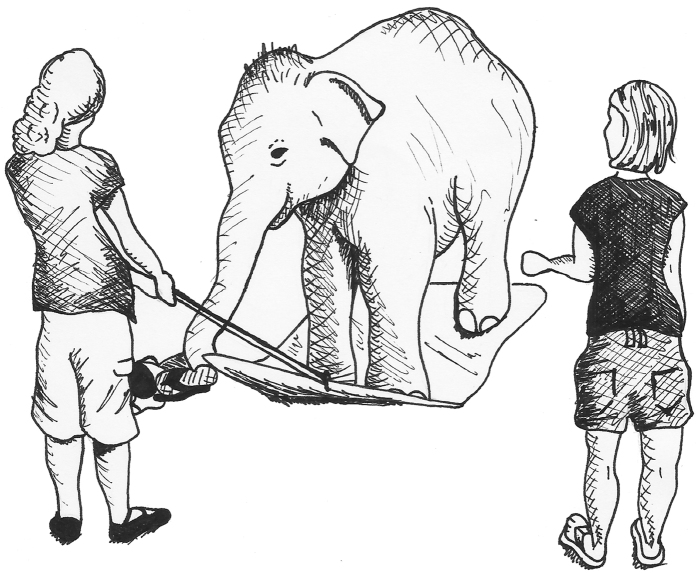
Set-up of the foot discomfort control. Once on the mat, elephants were requested to pick up flip flops and pass them to the experimenter whilst a second experimenter tugged on the rope at regular intervals. Drawing by E. Gilchrist.

To control for this, a second familiar experimenter pulled on the rope while subjects were undertaking a *different* task. We changed the task (from a stick exchange to an exchange of flip flops/footwear) to investigate the elephant’s general discomfort on the mat independent of the task itself. The task required subjects to pick up a pile of flip flops to their right and pass them to the experimenter on their left (Fig. 5).

Aside from these differences, the mat, rope and the commands used were the same as in the test condition, but subjects no longer had to move forward off the mat to exchange; they simply needed to move their head from right to left. Flip flops were used because subjects were accustomed to picking up human footwear during their daily work with tourists.

For elephants that completed this control condition after they had completed the test condition, the length of the trials mirrored each subject’s longest trial during the test condition (mean: 54.08 s, SD: 8.86 s). The rate at which the second experimenter pulled on the rope equalled each elephant’s mean tugging rate during test trials (mean: 11.02 tugs/minute, SD: 2.14). For the four subjects that underwent the control condition before they completed the test condition, an average was taken from all previously tested subjects (mean trial length: 54.25 s, SD: 2.36; mean : 11.4 tugs/minute, SD: 2.78).

### Analyses

All 12 subjects completed four sessions of 12 trials for each condition, with no more than two sessions being completed each day. An elephant succeeded in the test condition when they moved completely off the mat and pulled the stick and mat toward the experimenter. Success/failure was recorded live for every test trial. For both control conditions, we simply recorded whether or not subjects fully removed their weight from the mat in each trial.

Full results of factors in the final models of the GLMM analyses are presented in Table 1 and results of the random effects for each model are shown in Table 2. All statistics were run in R version 3.1.1 for Windows. The models used the glmer function of the lme4 package.

## Additional Information

**How to cite this article**: Dale, R. and Plotnik, J. M. Elephants know when their bodies are obstacles to success in a novel transfer task. *Sci. Rep.* **7**, 46309; doi: 10.1038/srep46309 (2017).

**Publisher's note:** Springer Nature remains neutral with regard to jurisdictional claims in published maps and institutional affiliations.

## Supplementary Material

Supplementary Movie S1

Supplementary Movie S1 Legend

Supplementary Dataset 1

## Figures and Tables

**Table 1:** Summary of generalized linear mixed models [GLMMs] to determine possible influences on subject’s likelihood to get off the mat.

	F	Df	p
Full model			
**Condition**	**230.42**	**2**	**<0.001**
Age group	0.17	1	0.66
	**χ^2^**	**Df**	**p**
Test vs. Stick-unattached: Session 1			
**Condition**	**82.08**	**1**	**<0.0001**
Test vs. Foot discomfort: Session 1			
**Condition**	**47.25**	**1**	**<0.0001**

The binary response variable was whether or not the subject removed their weight from the mat in each trial. Subject identity and order of conditions were included as random effects in the full model. Subject identity was included as a random effect in the model comparing the first sessions. Results in bold indicate significant effects.

**Table 2:** Variance and Standard Deviation [SD] for the random effects in each generalized linear mixed model [GLMM].

Random effect	Variance	SD
Full model		
Subject	0.42	0.65
Order	0.09	0.32
Test vs. Stick-unattached: Session 1		
Subject	0.62	0.83
Order	0.00	0.00
Test vs. Foot discomfort: Session 1		
Subject	1.34	1.16
Order	2.21	4.71
