# Supplementary material for: Elephants know when their bodies are obstacles to success in a novel transfer task
Source: Sci Rep. 2017 Apr 12;7:46309. doi: 10.1038/srep46309 (PMC5389349; doi:10.1038/srep46309)
Supplement: Supplementary Movie S1 Legend [file srep46309-s2.doc]

**Elephants know when their bodies are obstacles to success in a novel transfer task**

**Rachel Dale^1*^ and Joshua M. Plotnik^1,2,3,4&^**

1. Think Elephants International, Stone Ridge, NY, USA
2. Dept. of Psychology, University of Cambridge, Cambridge, U.K.
3. Mahidol University – Kanchanaburi, Sai Yok, Kanchanaburi, Thailand
4. Golden Triangle Asian Elephant Foundation, Chiang Saen, Thailand

*First author: [racheldale07@gmail.com](mailto:racheldale07@gmail.com), +43 69918332188

Current address: Messerli Research Institute, University of Veterinary Medicine, Veterinaerplatz 1, A-1210 Vienna, Austria

& Corresponding author: [Joshua.Plotnik@gmail.com](mailto:Joshua.Plotnik@gmail.com), +1 6462106138

Current address: Department of Psychology, Hunter College, City University of New York, 695 Park Avenue, New York, NY 10065

Supplementary Information

Movie Legend.

Movie S1. Video depicting each of the three conditions: test, stick-unattached control and foot discomfort control.
